# Pedigree reconstruction and spatial analysis for genetic testing and selection in a *Larix kaempferi* (Lamb.) Carrière plantation

**DOI:** 10.1186/s12870-022-03530-y

**Published:** 2022-03-28

**Authors:** Kyungmi Lee, In-Sik Kim, Kyu-Suk Kang

**Affiliations:** 1 Division of Tree Improvement and Biotechnology, Department of Forest Bio-Resources, National Institute of Forest Science, Suwon, 16631 Republic of Korea; 2 Department of Agriculture, Forestry and Bioresources, College of Agriculture and Life Sciences, Seoul National University, Seoul, 08826 Republic of Korea

**Keywords:** Genetic testing, Progeny trial, Pedigree reconstruction, Spatial autoregression, *Larix kaempferi*

## Abstract

**Background:**

*Larix kaempferi* is one of the major timber species in Northeast Asia. Demand for the reforestation of the species is rising in South Korea due to an increase in large timber production and utilization. However, progeny trials for the species have not been explored, making it challenging to foster advanced generations of tree improvement. In the present study, genetic testing and selection for diameter growth were conducted using pedigree reconstruction and phenotypic spatial distribution analysis in a plantation of *L. kaempferi*. The aim of the present study was to select the superior larch individuals using the pedigree reconstruction and phenotypic spatial distribution to substitute progeny trials. The plantation of seed orchard crops was established in 1990 and one-hundred and eighty-eight trees were selected as the study material. Genetic variation was investigated first to validate its adequacy as breeding material. Genetic testing was carried out using a model considering pedigree information and spatial autoregression of the phenotypes.

**Results:**

The expected heterozygosity of the mother trees and offspring were 0.672 and 0.681 presenting the corresponding level of genetic variation between two groups. The pedigree reconstruction using maternity analysis assigned one to six progenies to ninety-two candidate mothers. The accuracy of genetic testing was exceedingly increased with the animal model considering AR1 ⊗ AR1 structure compared to the animal model only. The estimated genetic variance of the former was 9.086 whereas that of the latter was 4.9E-5 for DBH. The predicted breeding values of the offspring for DBH were ranged from -5.937 cm to 5.655 cm and the estimated heritability of diameter growth was 0.344.

**Conclusions:**

The genetic testing approach based on pedigree reconstruction and phenotypic spatial distribution analysis was considered a useful analytical scheme that could replace or supplement progeny trials.

**Supplementary Information:**

The online version contains supplementary material available at 10.1186/s12870-022-03530-y.

## Background

The genus *Larix*, a deciduous tree species in the family Pinaceae, provides timber with high economic value as well as great ecological value in forests. Larch tree improvement programs have been carried out for the major timber species and hybrids in the genus [1–4]. *Larix kaempferi* (Lamb.) Carrière has been one of the major timber species in Korea since its introduction in 1904. Large scale plantations of the species were cultivated until the 1970s [5]. However, the plantation areas had since been reduced due the low timber production and difficulties encountered in larch wood processing for a time [6]. Conversely, demand for larch timber and seeds has been increasing since 2000s consistent with increase in timber production and developments in wood processing technologies [6]. Today, forestry policies that could expand the larch plantation and revitalize their use in Korea are being pursued in response to increasing demand. Over the past five years, the plantation area under *L. kaempferi* has increased considerably, contrary to the area under other major species. It reached 273,000 ha of forest area, which is the second largest after the area under *Pinus densiflora* (2015) [7].

Several studies have been conducted only on partial stages of the *L. kaempferi* tree improvement program in Korea. Provenance tests and adaptability tests had been performed to introduce the superior provenance of larch until 1987 [6]. 145 plus trees were selected during 1959–1980 and 306. 2 ha of seed orchard has been established to produce improved seeds of the species since 1968. [6]. In addition, studies to promote flowering and selection of multi-flowering clones have been carried out to address a key constraint in the unstable larch seed production [8, 9]. However, the tree improvement via progeny testing of the species was suspended due low levels of historic utilization, as well as a putatively limited gene pool caused by the introduction of only a small number of individuals to Korea.

Tree improvement is one of the essential activities in forest management, which is used to enhance the economic value of forests [10]. It aims to increase genetic gain in a gradual manner with recurrent testing and selection of advanced generations [11, 12]. The selection of advanced generation by genetic testing of larch has become necessary to ameliorate economic value of the species which has been actively used in plantation forestry in Korea. To shorten the time-consuming progeny trial approach of genetic testing, the alternative methods to the approach are required to meet current demand.

Pedigree reconstruction in plantations using seed orchard crops is one of the feasible approaches that could substitute the progeny trial method. Pedigree reconstruction analysis was first applied in tree improvement activities to detect the paternity arising from polymix breeding [13], followed by Breeding without Breeding [14], quasi-field trials [15], ad hoc breeding [16], and in situ genetic evaluation [17]. Pedigree reconstruction analysis is carried out to support existing tree improvement programs and to improve the accuracy of genetic parameter and breeding value estimates by substituting artificial crossing or wind pollination [18–22].

The accurate distinction of environmental effects is essential for proper genetic testing. Genetic testing considering spatial effects on the target traits has been attempted in previous studies in progeny trials or experimental forests [23]. Analytic models accounting for spatial structure could reduce the standard error associated with typical interfamily differences by 5–20% when compared to random complete block design experiments in genetic testing for tree height [24]. In addition, the fitness of the analysis was improved using the spatial model when compared to the incomplete block model in testing the growth properties of the *Pinus pinaster* [25]. The model has also been demonstrated to increase the accuracy of the log-likelihood and genetic value predictions based on restricted maximum likelihood [26]. The variation due to the spatial factors could be modelled effectively complementing the spatial autocorrelation. The supplementation of the spatial autocorrelation factor was more effective in the modeling of the spatial variation, significantly reducing the residual variation in a progeny trial of *Cunninghamia lanceolata* [23]. The genetic factor was efficiently explained considering the residuals caused by spatial autoregression of the target traits even in progeny trials based on a block design [26–30].

The objective of the present study was to perform genetic testing aided by pedigree reconstruction analysis and phenotypic spatial distribution analysis. The distinctive challenges of the present study to previous studies were the absence of a priori information on the family and the experimental design of the study site. The study was conducted in phases with two detailed subjects to analyze the genetic and environmental effects at the individual level, including 1) genetic variation analysis to determine the adequacy of the individuals as breeding material followed by pedigree reconstruction analysis, and 2) genetic testing based on pedigree information and phenotypic spatial autoregression. The overall aims of the present study were to present schematic of genetic testing in plantations that could be applied 1) to acquire basic genetic variation data and pedigree information on breeding materials of seed orchard crops and 2) to ameliorate the accuracy of genetic testing in plantations while considering phenotypic spatial distribution.

The following hypotheses were formulated: 1) the plantation of the offspring from the plus trees in the seed orchard would retain a significant amount of the genetic variation even though information about the introduced population is missing and 2) the genetic and environmental effects would be detectable from growth differences within and between the local groups in the plantation.

## Results

### Genetic variation in plantation of seed orchard crops of *Larix kaempferi*

The number of alleles and levels of heterozygosity in the three groups of 145 plus trees (PT) and offspring (HC and HS) were estimated using seven microsatellite markers. The average number of alleles (*N*_A_) and effective alleles (*N*_E_) were 9.7 and 3.6, respectively (Table 1). The estimated genetic parameters of the mother tree group were analyzed by the National Forest Seed and Variety Center [8]. Genetic variation in the *L. kaempferi* was slightly lower than that in the Japanese natural population (0.717–0.762) based on the average expected heterozygosity (*H*_E_) [31]. However, the difference was considered to be due to the low number of polymorphic markers used, as the estimates were in the 0.518–0.860 range. The inference was supported by the even lower *H*_E_ in the introduced 140 plus trees and the offspring in China with 17 microsatellite markers, at 0.525 and 0.557, respectively [32]. The plantation of the seed orchard crops, with the considerable genetic variation compared to that in the group of mother trees, was considered the appropriate base population.

**Table 1:** Genetic variation across populations of *Larix kaempferi* based on microsatellite marker analysis

Group	Locus	*N*_A_	*N*_E_	*H*_O_	*H*_E_	*F*
Plus trees	**Total**	**10.0** **(1.7)**	**3.7** **(0.7)**	**0.677** **(0.059)**	**0.672** **(0.058)**	**-0.010** **(0.027)**
Offspring (HC)	**Total**	**10.0** **(1.9)**	**3.7** **(0.7)**	**0.683** **(0.055)**	**0.681** **(0.050)**	**-0.006** **(0.035)**
	bcLK033	12.0	4.0	0.754	0.754	-0.002
	bcLK224	12.0	5.0	0.802	0.804	-0.001
	bcLK235	17.0	3.2	0.659	0.694	0.048
	bcLK241	4.0	2.1	0.572	0.526	-0.092
	LK4146	6.0	2.2	0.591	0.537	-0.104
	LK4170	14.0	7.0	0.908	0.860	-0.059
	LK4178	5.0	2.4	0.492	0.592	0.166
Offspring (HS)	**Total**	**9.1** **(1.4)**	**3.3** **(0.5)**	**0.669** **(0.053)**	**0.656** **(0.051)**	**-0.026** **(0.033)**
	bcLK033	12.0	4.6	0.723	0.785	0.075
	bcLK224	12.0	4.8	0.785	0.795	0.010
	bcLK235	11.0	3.1	0.645	0.683	0.052
	bcLK241	4.0	1.8	0.452	0.439	-0.034
	LK4146	6.0	2.1	0.577	0.529	-0.093
	LK4170	13.0	4.0	0.881	0.752	-0.177
	LK4178	6.0	2.6	0.621	0.612	-0.018
**Overall**	**Total**	**9.7** **(0.9)**	**3.6** **(0.3)**	**0.676** **(0.031)**	**0.671** **(0.029)**	**-0.014** **(0.018)**

*N*_A_: number of alleles; *N*_E_: effective number of alleles; *H*_O_: observed heterozygosity; *H*_E_: expected heterozygosity; *F*: Fixation index. The values in the parenthesis are the standard errors of the estimates

*N*_A_ and *N*_E_ were similar between PT and HC, with averages of 10.0 and 3.7, respectively (Table 1). The differences in *N*_A_ and *N*_E_ between PT and offspring (HC or HS) were not significant, with HS having slightly lower estimates of 9.1 and 3.3, respectively. Furthermore, neither *H*_O_ nor *H*_E_ were significantly different between PT and offspring. *H*_O_ in HC and HS were 0.683 and 0.669, respectively, showing little difference from the 0.677 in PT. *H*_E_ in the two offspring groups were 0.681 and 0.656, with no significant difference from 0.672 in PT. In a comparison between the two offspring groups (HC and HS), the differences in genetic variation were not significant, although some of the estimates were slightly lower in HS. The latter had lower *N*_*A*_, *N*_*E*_, *H*_*O*_, *H*_*E*_ values in except in the analysis with bcLK033 and LK4178. *N*_A_ in bcLK033 was similar between the two groups, but *N*_E_ was somewhat higher in HS. Both estimates of LK4178 were higher in HS than in HC, exhibiting distinctiveness from the other markers.

### Pedigree reconstruction of individuals in the *L. kaempferi* plantation

The average polymorphic information content (PIC) was 0.638 and the non-exclusion probability of identity was as low as 4.1E-07, based on the seven microsatellite markers. Each individual was identifiable as none of them showed the coincident genotypes in the seven loci. The non-exclusion probability of the first parent was 0.055. Ninety trees corresponding to 27% of the overall trees were assigned at relaxed confidence level (Table 2). The assignment of the other 244 trees was accomplished but did not fall within the confidence level. The proportions of assignments within the relaxed confidence level were 23% and 25% in HC and HS, respectively (Table 2). The low confidence level in the present study was considered to be derived from the characteristics of the used markers rather than the studied materials regarding the similar assignment patterns in the two discrete plantations.

**Table 2:** Maternity analysis of individual trees by population of *Larix kaempferi*

Level	Confidence (%)	Observed assignments (%)	
		Hongcheon	Hwaseong
Strict	95	10 ( 5%)	6 ( 4%)
Relaxed	80	53 ( 23%)	37 ( 25%)
Low	< 80	135 ( 71%)	109 ( 75%)
Total		188 (100%)	146 (100%)

The maternity analysis assigned the offspring to 92 and 79 candidate mothers in HC and HS respectively (Table S1). The total number of the candidate mothers was 112. The number of mutually exclusive mother trees was 33 in HC and 20 in HS, displaying the diverse constitution of the mother trees. The results potentially indicated that the genetic constitution would be distinguished by the reproduction year considering the samples of the seed orchard crops were produced in different years. The number of progenies by each mother tree were 1–6 trees in HC. The mother trees with six progenies were CN05, JPR-4, and CN09, and the 42 mother trees had only the one offspring. The number of progenies by each mother tree were 1–5 trees in HS. The assignment revealed that the mother trees GB12 and GB27 each had five progenies, and 36 mother trees had one offspring in HS. The offspring by mother trees were relatively evenly dispersed in the plantation (Fig S1–S5). Similarly, majority of the mother trees had one offspring according to the results of pedigree reconstruction analyses for *Abies nordmanniana* and *Larix occidentalis* [15, 17].

### Genetic testing in the plantation of *L. kaempferi*

The prediction of breeding values was performed for each mother tree and the individual tree in the plantation although the confidence level of the maternal assignment was rather low. The estimated variance of the genetic effect was infinitesimal, at 4.9E-05, and the estimate for the residual was 25.07. Consequently, differences among the individuals were imperceptible (Table S2). The maximum predicted breeding value was 0.00003 cm for hc1485, with a DBH 29.7 cm, and the minimum estimates was -0.00002 cm for hc1442, with a DBH of 6.6 cm. The correlation coefficient of the observed and predicted value was 0.94, which could be explained with predictions considering not only observations but also maternal information.

**Fig. 1:**
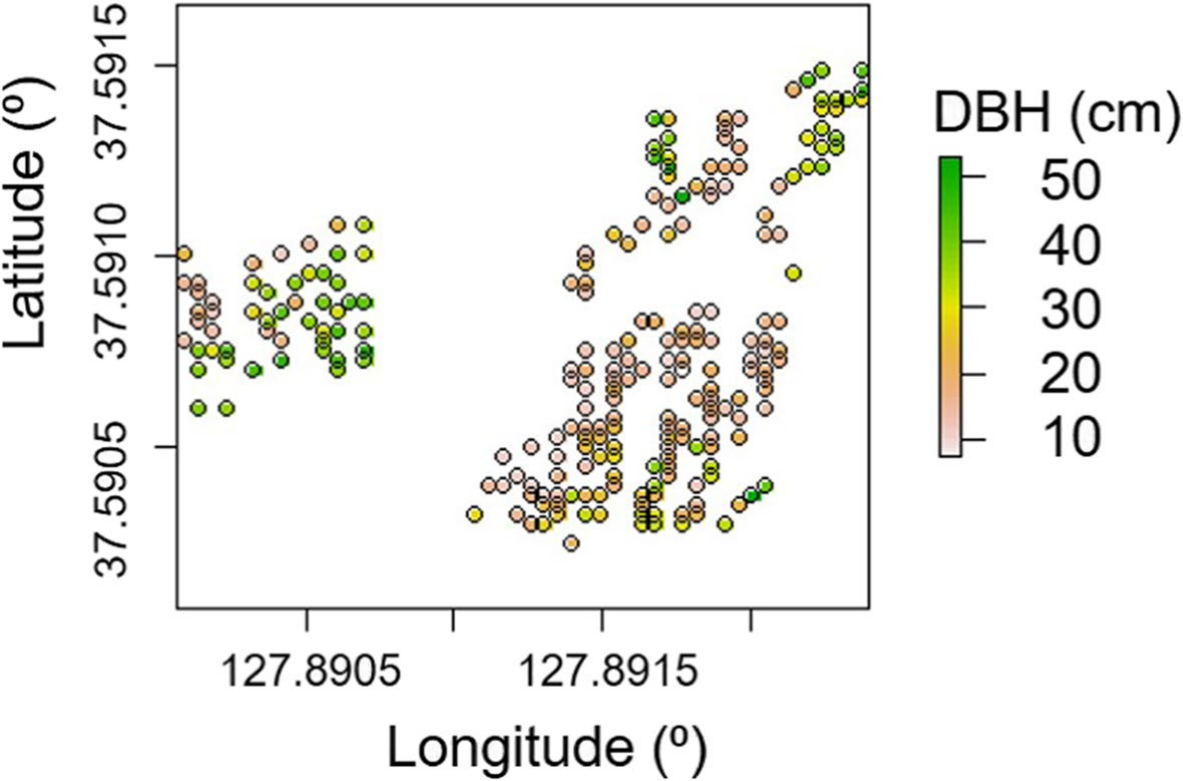
Rasterization of DBH distribution for the spatial analysis using AR1 ⊗ AR1 in *Larix kaempferi* stand

To separate the genetic effects with increased accuracy, the environmental effects were investigated to be accounted in the model of genetic testing. The spatial autocorrelation of phenotype was significant using Moran’s I index (*p* < 7.4E-12). The results of seven types of spatial models, including exponential, gaussian, spherical, linear, rational, AR1, AR1 ⊗ AR1, and the basic animal model were compared to assess the model that would most adequately explain the phenotypic spatial structure of the plantation. The distribution of the phenotype was rasterized for use in the spatial analysis with AR1 ⊗ AR1 (Fig. 1). The rasterization was cautiously processed to prevent data loss due to adjacency between individuals, considering their irregular distribution.

**Fig. 2:**
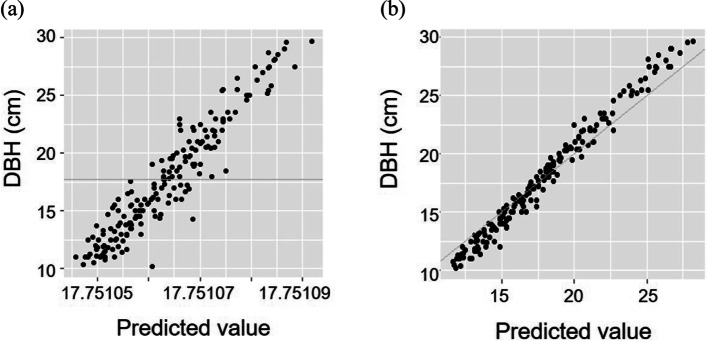
Fitness plot based on (a) animal model and (b) animal + AR1 ⊗ AR1 model analysis

**Fig. 3:**
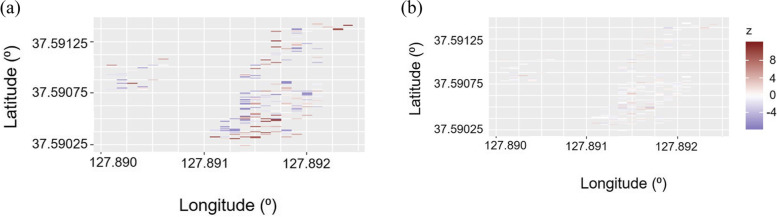
Comparison of (a) residual of animal model and (b) residual of animal + AR1 ⊗ AR1 model

The integration of the animal and AR1 ⊗ AR1 model (animal + AR1 ⊗ AR1) yielded the highest fitness followed by the basic animal model, based on the log likelihood and AICc (Table 3). The AICc of the animal + AR1 ⊗ AR1 model was largely distinct from those of the other models, highlighting its superior fitness. The fitness of the model with spatial AR1 was very low, suggesting the need for the use of a two-dimensional first-order autoregression model. The fitness and residual of the animal model and animal + AR1 ⊗ AR1 model were confirmed in the present study (Fig. 2, Fig. 3). The residual was reduced in the animal + AR1 ⊗ AR1 model when compared with in the animal model.

**Table 3:** Comparison of the fitness of spatial autoregression models of DBH distribution in *Larix kaempferi* stand

**Spatial model**	**Intercept**	**Degree of freedom**	**Log likelihood**	**AICc**^1)^
AR1 ⊗ AR1	18.07	3	-553.6	1113.1
Animal model	17.751	2	-568.9	1141.7
Rational	17.8	5	-568.1	1146.5
Gaussian	17.76	5	-568.1	1146.6
Spherical	17.76	5	-568.3	1147.0
Exponential	17.8	5	-568.4	1147.2
Linear	17.76	5	-568.4	1147.2
AR1	18.18	4	-603.9	1216.0

^1)^Second-order Akaike information criterion

**Fig. 4:**
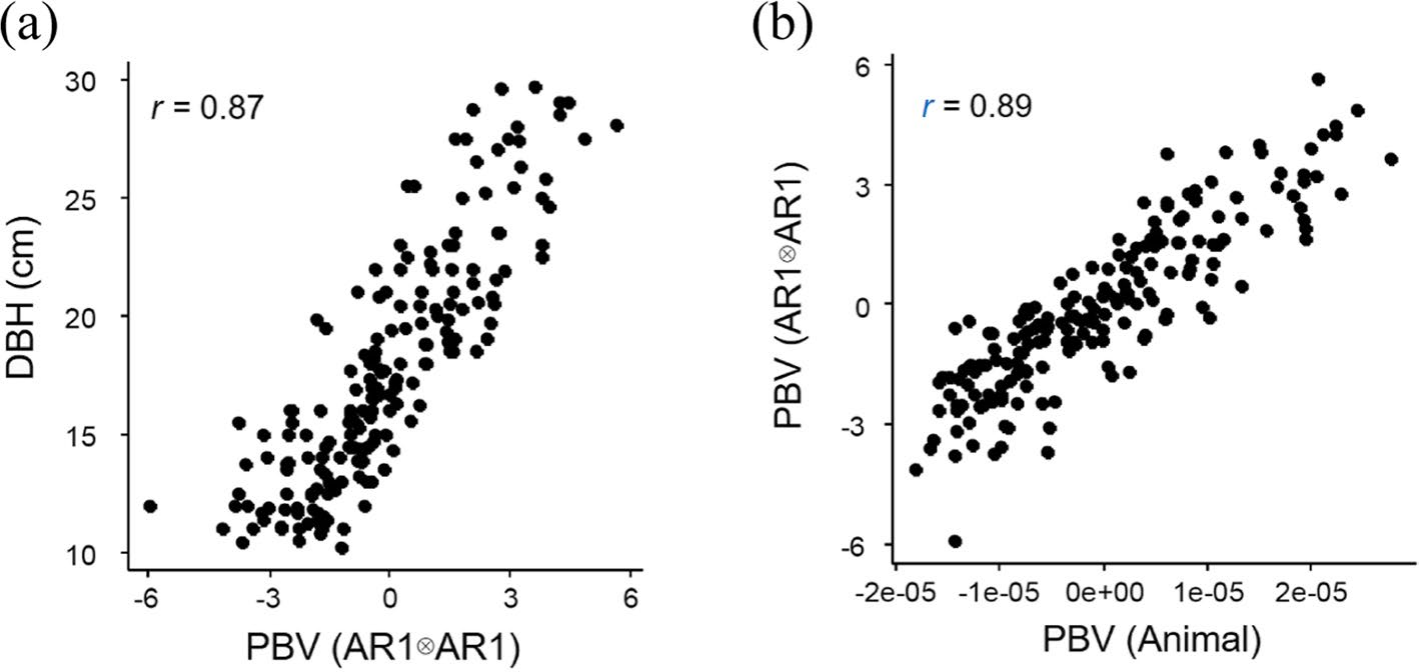
Correlation between (a) DBH and the predicted breeding value (PBV) based on animal + AR1 ⊗ AR1 model, (b) predicted breeding values based on animal model and animal + AR1 ⊗ AR1 model

The variance component of genetic factor was 9.086, and the heritability was estimated as 0.344 (Table 4). The accuracy of the genetic testing improved following the elimination of the environmental effects, with a heritability estimate of 0.224 for *L. kaempferi* DBH [1]. The predicted breeding value (PBV) of each individual also reflected the enhanced power of explanation of the animal + AR1 ⊗ AR1 model (Table S2). The maximum and minimum PBV were 5.655 cm and -5.937 cm, respectively, showing the apparent difference between the animal model and the animal + AR1 ⊗ AR1 model. The Spearman correlation coefficient (*r*) between the observation and the predicted breeding value using the animal + AR1 ⊗ AR1 model was 0.87 (Fig. 4). The weakened correlation was considered to be due to the consideration of the environmental effects when evaluating the phenotypes. The *r* between the prediction by the animal model and the animal + AR1 ⊗ AR1 model was 0.89, revealing the dissimilar estimates obtained by the two models. The breeding values predicted by the two models were compared to confirm the frequent changes in rank (Table S2) reflecting the practicality of spatial analysis approaches in genetic testing.

**Table 4:** Variance components based on animal + AR1 ⊗ AR1 model analysis of DBH growth of *Larix kaempferi*

	**Estimated variances**	**Standard error**
Genetic	9.086	7.634
Spatial	12.478	3.894
Residual	4.885	7.393

**Fig. 5:**
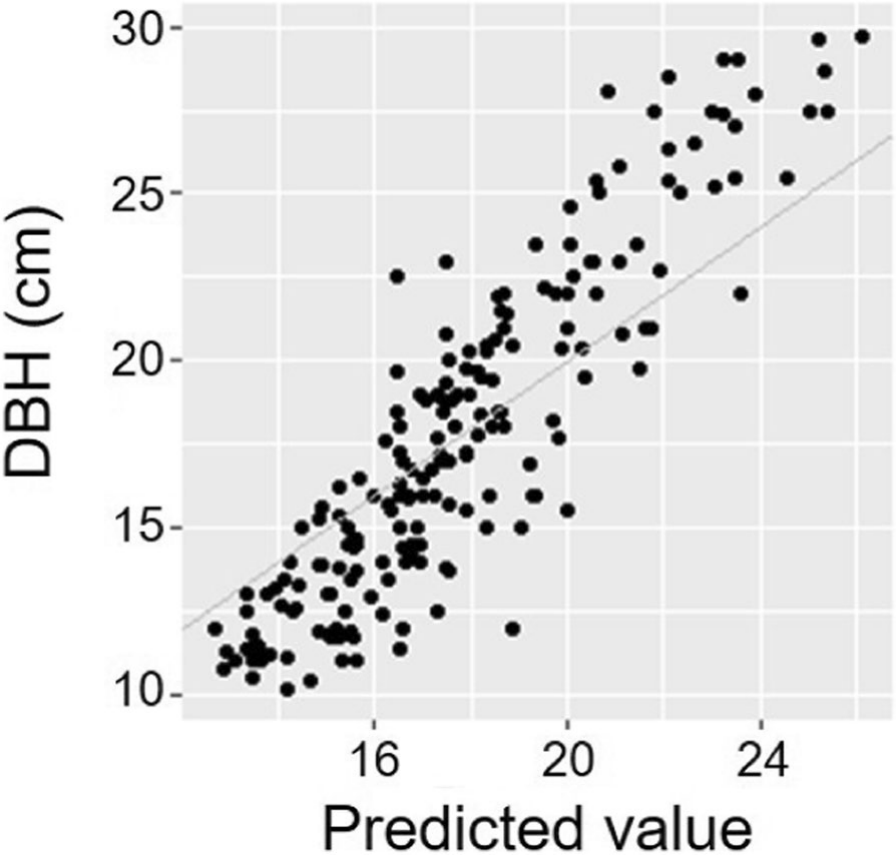
Fitness plot by animal + AR1 ⊗ AR1 model analysis with family effect

PBVs of mother trees were estimated from the fitted model (Fig. 5), with the highest value obtained in KW02, followed by JB10, NO05, NO03, and GB25 (Table S3). The PBV of GB23 was the lowest followed by JB08, CN15, and KW56. In the case of KW02, which had the highest PBV, five trees were assigned to its family in HC (Table S1, Table S3). All the five individuals, including hc1327, hc1427, hc1485, hc1538, and hc1549, had positive PBVs of 1.507 cm, 4.846 cm, 3.621 cm, 2.171 cm, and 0.117 cm, respectively. JB10, which had the second highest PBV, was the mother tree of hc1335, which showed the highest PBV among the offspring, at 5.655 cm. The progenies of NO05 were hc1436 and hc1552, with PBVs of 3.971 cm and 2.604 cm, which were the 6th and 24th highest PBVs among the 188 trees. NO03 had two progenies (hc1437 [PBV: 3.795 cm], hc1463 [PBV: 2.442 cm]), and GB25 had five progenies (hc1423 [PBV: 2.390 cm], hc1468 [PBV: -1.163 cm], hc1519 [PBV: 0.875 cm], hc1520 [PBV: 3.900 cm], and hc1559 [PBV: 2.390 cm]). GB23, the mother tree with the lowest PBV, had two progenies, hc1509 and hc1516, with PBVs of -3.812 cm and -5.937 cm, respectively. The latter had the lowest breeding value among the 188 trees. The PBVs of mother trees were considered to reflect the PBVs of their progenies, however the accuracy of analysis was limited due to the insufficient number of mother trees and their progenies.

## Discussion

### Genetic variation in plantation of seed orchard crops of *Larix kaempferi*

Forest trees, which are long-lived plants that undergo outcrossing frequently, generally have high genetic diversity, excluding in the cases of endangered or rare species. The *L. kaempferi* seed orchard offspring was confirmed to retain considerable genetic variation in the present study. High genetic diversity was detected in the limited natural distribution range of *L. kaempferi* in Mt. Fuji even on fragmented habitats [31]. Frequent gene flow between populations was inferred from the low level of genetic differentiation despite the geographical distance in both Japanese larch and European larch [31, 34].

Studying the change in the genetic variation using seed orchard crops is necessary to detect and preclude decline in genetic diversity with increased inbreeding [35–37]. Similar levels of genetic diversity in seed orchards and their crops are frequently reported, dispelling concern over narrowed genetic base in tree improvement programs. The genetic diversity of the progeny trial corresponded to that of a naturally regenerated stand in *Picea sitchensis* and *Picea abies* [35, 36]. In addition, the genetic diversity of a plantation with seed orchard crops was not affected by a tree improvement program in *Cryptomeria japonica* [37].

However, genetic variation in larch should be assessed cautiously. Genetic variation in larch is influenced by the mating system, reproductive characteristics, and changes in descendants. Deficit of heterozygosity has been detected in *Larix sibirica* and *Larix gmelinii*, and attributed to inbreeding [38]. Furthermore, in a study on the larch mating system, the distance of pollen dispersal was reported to be relatively short [32, 39, 40], and the genetic structure of European larch over its entire distribution was more obvious than those of other conifers [41].

In summary, larch generally retain considerable genetic variation, similar to most forest trees. However, the species could be experience decreased genetic variation due to inhibited gene flow or severe fluctuation in flowering and seed production activities [6, 8, 42].

The first prerequisite in tree improvement is understanding the genetic variation in the natural population and in the selected breeding population [43]. Genetic variation is the key factor influencing natural and artificial selection activities [44] related with the coefficient of variance of the genetic factor. Genetic variation determines the genetic gain and the potential of improvement by influencing adaptability and resistance to biotic or abiotic stress [45]. Studying tree genetic variation is also essential for sustainable forest management and conservation [46], which could enhance the understanding of variation in adaptable traits including growth, wood characteristics, and resistance to disease or drought [47]. Understanding genetic variation is particularly essential prior to genetic testing in *L. kaempferi* plantation considering the reproduction and mating characteristic of the species. The additional introduction to enrich the gene pool of larch would assure the genetic diversity of breeding materials in long-term tree improvement program.

### Pedigree reconstruction of individuals in the *L. kaempferi* plantation

Using an increased number of polymorphic markers would be necessary to enhance the confidence level of the maternity assignment in *L. kaempferi*. A study investigated the accuracy of pedigree reconstruction using a microsatellite marker as the principal marker in the field of research [48]. The success rate of pedigree reconstruction was improved with an increase in the number of markers in the pedigree reconstruction analysis for European sturgeon (*Acipenser sturio*) [49]. The low confidence level of parental assignment was thought to be related with allele frequency in a *Abies procera* study, where the microsatellite marker revealed insufficient variation [48]. The separate experiment and genotyping of the plus trees and the offspring was the other plausible reason of a decrease in the confidence in this study. The simultaneous analysis of the overall sample would reduce the scoring error of genotyping [50].

The application of molecular biology technologies in tree improvement can be classified into two approaches [12, 48], including association between target traits and markers, such as the genetic dissection or genomic selection, and enhancing tree improvement efficiency using DNA markers, which includes pedigree reconstruction analysis [51, 52]. The development of the DNA markers with high resolutions increases the usefulness of the pedigree reconstruction [53]. Furthermore, it is expected that the confidence of assignment would be enhanced by the rapid development and commercialization of automated and high-throughput genotyping approaches, including SNP marker analysis [48].

The small number of assigned progenies by parent has been discussed as one of the main challenges of genetic testing with pedigree reconstruction [15], and it impedes researchers’ ability to obtain sufficient numbers of samples required to establish statistical significance in genetic testing. Therefore, forward selection is considered desirable compared to the backward selection in the case. The latter has been recommended considering the relative ease of propagation following tree improvement acceleration [16]. However, forward selection was suggested in this study due to the possibility of deterioration of accuracy when performing genetic testing on mother trees.

### Genetic testing in the plantation of *L. kaempferi*

Genetic gain following selection is expected in the genus *Larix* considering previous genetic testing [2, 17, 54–57]. The heritability of *L. decidua* height was 0.23 in the 25–35 age group [17], and the heritability values for height and DBH estimates of *L. kaempferi* were 0.549 and 0.224, respectively in the genetic testing of artificial crosses [1]. The infinitesimal genetic variance in the analysis with the animal model highlighted the need for isolating the environmental factor. The plantation was located in a highly heterogeneous site, which hampered the analysis of environmental effects. Prior pedigree reconstruction studies have mainly been carried out in experimental forests under intensive management, with a focus on the development of analytical methodologies [14–17, 21, 22, 58]. In addition, few substantive genetic testing studies have been conducted in the genus *Abies* [15, 16, 48], and the genetic resources of *L. decidua* have been evaluated based on locations [17]. The effective analysis of environmental effects is a major obstacle in genetic testing. Notably, the application of pedigree reconstruction in practical genetic testing has been accomplished in the study of genus *Abies* by supplementation with polymorphic SNP marker and spatial analyses.

The individual trees and their mother trees were evaluated with PBVs based on the animal + AR1 ⊗ AR1 model in this study. Considering the spatial autoregression as the products of innumerous biotic and abiotic factors proved useful in the plantation genetic testing, in combination with the pedigree reconstruction analysis. The inferior fitness of the spatial model except AR1 ⊗ AR1 highlighted the importance of selecting the adequate spatial structure model beyond the consideration of the existence of spatial autocorrelation. The enhancement of genetic testing via spatial analysis with AR1 ⊗ AR1 has been reported [26–30]. Genetic testing with the model considering AR1 ⊗ AR1 structure in pedigree reconstruction analysis has been used in family selection in a *Abies bornmülleriana* progeny trial [16].

It is impossible to control the number of samples by family in genetic testing in plantations, contrary to the case in traditional progeny testing. Only one progeny was assigned to the multiple family in the present study as in the pedigree reconstruction analysis in *L. occidentalis* and *A. nordmanniana* [15, 17]. The spatial analysis was considered to be essential in the selection without the experimental design including the replication of the family. Spatial analysis is also considered to be useful in the progeny trials [23, 27, 29], in particular, under high environmental heterogeneity, for example in mountainous sites in Korea [59].

## Conclusions

Genetic testing was conducted in a *L. kaempferi* plantation to establish a systematic scheme that could supplement progeny trials in tree improvement activities. Pedigree reconstruction and phenotypic spatial distribution were applied in the analysis of genetic and environmental effects on tree growth. The pedigree reconstruction was performed for individual trees following the assessment of genetic variation to identify the adequacy of breeding population. The genetic testing was implemented considering the spatial autoregression to dissect the environmental factors influencing the growth characteristics of a plantation without experimental design.

Several factors should be taken into account in the course of interpreting the results of the present study. The planting and management records of the study site would save the additional labor for the verification of the data. In addition, assessing the genetic diversity is necessary for preventing a decrease in genetic variation and inbreeding depression in advanced generations. Furthermore, appropriate selection strategies should be adopted based on the limited sample sizes obtained in the progeny. Forward selection was suitable in the present study considering the small progeny size per mother tree. The effective application of the pedigree reconstruction in genetic testing requires the combined analysis of phenotypic spatial distribution as well as the influence of numerous environmental factors.

## Materials and Methods

### Study sites and sampling

The *L. kaempferi* plantation of seed orchard crops, owned by the state, was selected as the study site based on plantation history. The reforestation was undertaken on the preexisting larch plantation in Hongcheon-gun (HC), Gangwon-do (37°35′24.0'N, 127°53′24.0'E) with 3,000 larch seedlings / ha over an area of 1.3 ha in 1990. The remaining number of larch trees at present was 271 including the preexisted old trees. Therefore, the study materials were selected through screening to take the field circumstances into consideration preceding the analysis and in accordance with the national forest management record. The study complied with the guidelines of the National Institute of Forest Science (NIFoS) and the legislation of plant ethics in South Korea.

The growth data and the empirical yield table were inspected in the screening. The average height and DBH of 271 trees in the plantation were 16.0 m and 23.7 cm. The respective average values were 14.4 m and 18.7 cm under the lowest site index (16) and 19.5 m and 21.8 cm under the highest site index (22) based on the empirical yield [60]. The outlying 83 trees with DBH > 30 cm were excluded from further analyses.

**Fig. 6:**
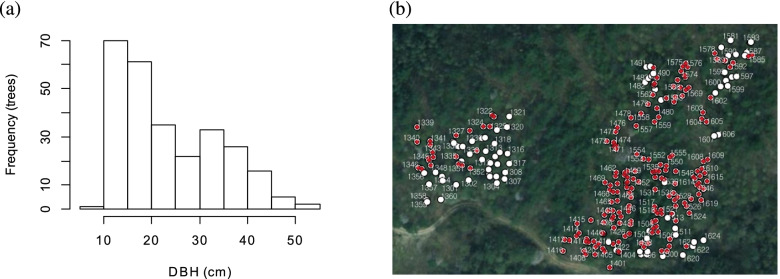
(a) Histogram of the diameter at breast height (DBH) of *Larix kaempferi* individuals in the studied stand (b) The 188 individual trees selected for analysis (red marks) and the excluded trees (white) in the *Larix kaempferi* plantation

The overlapping existence of different age group of trees was sustained by deviation from the normal distribution pattern of the growth data (Fig. 6). The rest of the 188 were considered to be the subsequent generation from the seed orchard based on the scarce chance of larch seedling occurrence in the larch forest. The screening based on DBH was possible in the present study because the differences in growth between the excluded and remnant trees were rather distinct. One-hundred and forty-six trees from a five-year-old seed orchard planted in Hwaseong-si (HS), Gyeonggi-do, were additionally included in the analysis for the comparison with the HC plantation.

Three types of data were collected for the phenotype, genotype, and spatial distribution analyses of the individuals. Tree height and DBH, cambium or leaf samples, and GPS coordinates (Trimble Geo 7X, Trimble Inc., Sunnyvale, CA, USA) were collected simultaneously.

### Genetic variation and pedigree reconstruction of *L. kaempferi* plantation

The isolated DNA of 334 *L. kaempferi* trees were genotyped using seven microsatellite markers, which were used in a previous analysis on the mother trees in the seed orchard [61, 62] (Table 5). The genotype data of the mother trees used in the present study were assessed by the National Forest Seed and Variety Center (NFSV). The polymerase chain reaction (PCR) was carried out using FAM-labelled forward primers of each marker followed by genotype analysis using an ABI 3730xl Genetic analyzer and GeneMapper 4.0 (Applied Biosystems, Foster City, CA, USA).

**Table 5:** Microsatellite markers of *Larix kaempferi* used in the present study

Marker	Primer name	Primer Sequence (5’ → 3’)
bcLK033	bcLK033-F	GGAAATGTAGAGATGAGCAATAA
	bcLK033-R	AGGTGCGGTAGTACAAAGTGA
bcLK224	bcLK224-F	GGAGAGGCCACTACTATTATTAC
	bcLK224-R	ATGCGTTCCTTCATTCCTCT
bcLK235	bcLK235-F	TTCACTTGTGATCCTAGAGTTAGA
	bcLK235-R	AACCCCTAACCATATAATATCCA
bcLK241	bcLK241-F	TGAGGTTAGGAGCATCTCGT
	bcLK241-R	GTCCTTCATCGCCTCTTCTT
LK4146	LK4146-F	CAACATGTTTCTCCTACCACCA
	LK4146-R	TCAGACATTCCCAAACATGC
LK4170	LK4170-F	TTTTCCAAAGCCAAAATTCTACA
	LK4170-R	TATGAGCCCGACCCTATTTG
LK4178	LK4178-F	TCCACCTTAGCACTCCCACT
	LK4178-R	GGGGCCTTTATAGGTTGGTT

Genetic variation in the larch plantation of seed orchard crops was estimated based on the number of alleles (*N*_A_), the number of the effective alleles (*N*_E_), observed heterozygosity (*H*_O_), expected heterozygosity (*H*_E_), and fixation index (GenAlEx 6.5) [63]. The data on the mother trees were provided by the NFSV [6].

The maternity analysis was conducted for the pedigree reconstruction of the *L. kaempferi* plantation. The individuals in the plantation were assigned to the mother trees in the seed orchard using CERVUS 3.0 based on the maximum likelihood approach [64, 65]. The confidence levels were set at 95% (restricted) and 80% (relaxed), with default values in the program. The error rate of genotype identification was specified as 0.01 and the confidence level was decided based on 10,000 simulations.

### Genetic testing using pedigree reconstruction and spatial distribution of phenotype

The growth correlations among adjacent individuals were observed in the study site. Spatial autocorrelation was tested based on DBH using Moran’ I prior to genetic testing. As the existence of the spatial autocorrelation of DBH was identified, the spatial model was explored using seven spatial structures, including AR1, exponential, Gaussian, spherical, linear, rational, and AR1 ⊗ AR1 [66, 67] to verify the utility of AR1 ⊗ AR1. The best fitted model was selected by comparing the log-likelihood values and the second-order Akaike Information Criterion (AICc) of the eight linear mixed models, including the animal model [68, 69]. The rasterization of the plantation was conducted to form 450 rows and 450 columns for AR1 ⊗ AR1 analysis. The accuracy of the genetic testing was improved using the AR1 ⊗ AR1 model, which is the Kronecker product of the autoregression matrix for rows and columns [70, 71].

y = X*b* + Z*u* + ξ + η.

where y is the vector of data; *b* is a vector of fixed effects; *u* is a vector of random effects (genetic or family effect); ξ is spatially dependent residuals with a variance–covariance matrix of ${\sigma }_{\upxi }^{2}$[AR1(*ρ*_col_) ⊗ AR1(*ρ*_row_)] and η is spatially independent residuals (nugget effects). AR1(*ρ*_col_) and AR1(*ρ*_row_) are the first-order autoregressive correlation matrices in column and row respectively.

The breeding values of mother trees and the individuals in the plantation were predicted by the best fitted model using R program [72]. Existence of spatial autocorrelation was tested with package ape [73] and the rasterization was conducted using sp [74] and raster [75]. The analyses of models with spatial structures were conducted with package nlme [66], breedR [67] compared using MuMIn [76]

## Supplementary Information

**Additional 1: Table S1. **Number of progenies assigned to the plus trees by maternity analysis **Table S2.** Comparison of the predicted breeding value(PBV) and its rank of individual based on Animal model and Animal+AR1⊗AR1 model **Table S3.** Predicted breeding values (PBV) of *Larix kaempferi* trees assigned as mother trees **Fig S1.** Locations of the progenies of mother trees with six assigned progenies **Fig S2.** Locations of the progenies of mother trees with (a) five assigned progenies, and (b) four assigned progenies. **Fig S3.** Locations of the progenies of mother trees with three assigned progenies **Fig S4.** Locations of the progenies of mother trees with two assigned progenies **Fig S5.** Location of the progenies of mother trees with one assigned progeny.

## Data Availability

The datasets used and/or analyzed during the current study are available from the corresponding author on reasonable request.
