# Supplementary material for: Pedigree reconstruction and spatial analysis for genetic testing and selection in a Larix kaempferi (Lamb.) Carrière plantation
Source: BMC Plant Biol. 2022 Mar 28;22:152. doi: 10.1186/s12870-022-03530-y (PMC8962119; doi:10.1186/s12870-022-03530-y)
Supplement: Supplementary file 1 — Additional 1: Table S1. Number of progenies assigned to the plus trees by maternity analysis Table S2. Comparison of the predicted breeding value(PBV) and its rank of individual based on Animal model and Animal+AR1⊗AR1 model Table S3. Predicted breeding values (PBV) of Larix kaempferi trees assigned as mother trees Fig S1. Locations of the progenies of mother trees with six assigned progenies Fig S2. Locations of the progenies of mother trees with (a) five assigned progenies, and (b) four assigned progenies. Fig S3. Locations of the progenies of mother trees with three assigned progenies Fig S4. Locations of the progenies of mother trees with two assigned progenies Fig S5. Location of the progenies of mother trees with one assigned progeny. [file 12870_2022_3530_MOESM1_ESM.docx]

Table S1. Number of progenies assigned to the plus trees by maternity analysis

| **Plus tree** | **Hongcheon** | **Hwaseong** | **Plus tree** | **Hongcheon** | **Hwaseong** |
| --- | --- | --- | --- | --- | --- |
| CB01 | - | 3 | GB23 | 2 | - |
| CB03 | 1 | - | GB24 | 1 | 1 |
| CB06 | 1 | - | GB25 | 5 | 1 |
| CN04 | 1 | - | GB26 | - | 2 |
| CN05 | 6 | 1 | GB27 | 3 | 5 |
| CN08 | - | 1 | GB28 | - | 1 |
| CN09 | 6 | 4 | GB29 | 2 | - |
| CN10 | 2 | 1 | GB31 | 2 | - |
| CN13 | 1 | - | GG01 | 2 | 1 |
| CN14 | 3 | 3 | JB01 | 3 | 4 |
| CN15 | 2 | 3 | JB03 | 3 | 1 |
| CN16 | 2 | 1 | JB05 | 2 | - |
| CN18 | - | 1 | JB07 | - | 2 |
| CN19 | 1 | 2 | JB08 | 3 | 2 |
| GB04 | 1 | - | JB09 | 2 | 2 |
| GB05 | 1 | - | JB10 | 1 | 3 |
| GB07 | - | 3 | JB11 | 1 | - |
| GB09 | 2 | - | JB12 | 1 | 1 |
| GB10 | - | 2 | JB13 | 1 | 1 |
| GB11 | 1 | - | JB14 | 3 | - |
| GB12 | 1 | 5 | JB15 | 1 | - |
| GB13 | 1 | 2 | JJ01 | 1 | - |
| GB14 | - | 1 | JN01 | 3 | 4 |
| GB15 | - | 1 | JN03 | 1 | 1 |
| GB16 | 1 | - | JN04 | 5 | 1 |
| GB18 | - | 2 | JPOR-1 | 1 | - |
| GB19 | - | 1 | JPR-1 | 3 | 1 |
| GB21 | 2 | 1 | JPR-10 | 2 | - |
| JPR-12 | 3 | 2 | KW28 | 2 | 3 |
| JPR-3 | 2 | 1 | KW29 | 2 | 2 |
| JPR-4 | 6 | 2 | KW30 | 2 | - |
| JPR-5 | - | 2 | KW31 | 1 | - |
| JPR-8 | 1 | 4 | KW33 | - | 1 |
| JPR-9 | 2 | - | KW34 | 4 | 2 |
| KW01 | - | 1 | KW35 | 1 | 2 |
| KW02 | 5 | 2 | KW38 | 1 | 1 |
| KW03 | 4 | 2 | KW40 | 1 | 1 |
| KW04 | 1 | 1 | KW42 | 3 | 1 |
| KW06 | 1 | 1 | KW43 | 2 | 1 |
| KW07-1 | 3 | 2 | KW44 | 1 | - |
| KW07-2 | 3 | 4 | KW47 | - | 1 |
| KW08 | 4 | 3 | KW48 | 1 | 1 |
| KW09 | 3 | 2 | KW51 | 1 | - |
| KW10 | 3 | 2 | KW52 | 2 | 1 |
| KW11 | - | 1 | KW54 | 1 | - |
| KW12 | 1 | - | KW55 | 1 | 2 |
| KW13 | 2 | 2 | KW56 | 4 | - |
| KW15 | 1 | 1 | KW57 | 1 | 3 |
| KW16 | - | 1 | KW58 | 2 | - |
| KW19 | 3 | 2 | KW59 | 1 | 2 |
| KW20 | 1 | - | NO01 | - | 1 |
| KW21 | 1 | - | NO02 | 1 | 2 |
| KW22 | 1 | 1 | NO03 | 2 | - |
| KW23 | 1 | - | NO04 | - | 3 |
| KW24 | 3 | 3 | NO05 | 2 | 1 |
| KW26 | 1 | 1 | NO06 | 3 | - |

Table S2. Comparison of the predicted breeding value (PBV) and its rank of individual based on Animal model and Animal+AR1⊗AR1 model

| **Tree** | **Animal + AR1⊗AR1 model** | | **Animal model** | | **DBH** | **Rank** |
| --- | --- | --- | --- | --- | --- | --- |
| **ID** | **PBV** | **Rank** | **PBV** | **Rank** |  | **(DBH)** |
| hc1335 | 5.655 | 1 | 2.06E-05 | 7 | 28.1 | 7 |
| hc1427 | 4.846 | 2 | 2.44E-05 | 2 | 27.5 | 9 |
| hc1429 | 4.466 | 3 | 2.24E-05 | 5 | 29 | 3 |
| hc1410 | 4.252 | 4 | 2.24E-05 | 4 | 29 | 4 |
| hc1515 | 4.236 | 5 | 2.12E-05 | 6 | 28.5 | 6 |
| hc1436 | 3.971 | 6 | 1.50E-05 | 21 | 24.6 | 25 |
| hc1520 | 3.9 | 7 | 1.99E-05 | 9 | 25.8 | 17 |
| hc1453 | 3.799 | 8 | 1.17E-05 | 25 | 23 | 29 |
| hc1437 | 3.795 | 9 | 1.51E-05 | 20 | 25 | 23 |
| hc1450 | 3.784 | 10 | 6.05E-06 | 51 | 22.5 | 34 |
| hc1485 | 3.621 | 11 | 2.77E-05 | 1 | 29.7 | 1 |
| hc1478 | 3.266 | 12 | 1.71E-05 | 17 | 26.3 | 16 |
| hc1562 | 3.229 | 13 | 1.92E-05 | 14 | 27.4 | 13 |
| hc1406 | 3.186 | 14 | 2.04E-05 | 8 | 28 | 8 |
| hc1477 | 3.082 | 15 | 1.04E-05 | 32 | 25.4 | 20 |
| hc1559 | 3.068 | 16 | 1.93E-05 | 12 | 25.4 | 21 |
| hc1593 | 2.949 | 17 | 1.67E-05 | 18 | 27.5 | 10 |
| hc1563 | 2.847 | 18 | 8.90E-06 | 38 | 21.9 | 42 |
| hc1585 | 2.778 | 19 | 2.28E-05 | 3 | 29.6 | 2 |
| hc1430 | 2.743 | 20 | 8.10E-06 | 42 | 23.5 | 26 |
| hc1497 | 2.698 | 21 | 1.82E-05 | 16 | 27 | 14 |
| hc1339 | 2.688 | 22 | 1.28E-05 | 24 | 23.5 | 27 |
| hc1618 | 2.65 | 23 | 8.90E-06 | 37 | 21.5 | 43 |
| hc1552 | 2.604 | 24 | 8.90E-06 | 36 | 20.5 | 52 |
| hc1612 | 2.563 | 25 | 6.08E-06 | 50 | 20.8 | 49 |
| hc1536 | 2.531 | 26 | 3.89E-06 | 65 | 19.7 | 61 |
| hc1463 | 2.442 | 27 | 6.11E-06 | 48 | 19 | 67 |
| hc1423 | 2.39 | 28 | 1.90E-05 | 15 | 25.2 | 22 |
| hc1537 | 2.208 | 29 | 7.55E-06 | 43 | 20.6 | 51 |
| hc1538 | 2.171 | 30 | 1.10E-05 | 28 | 18.5 | 74 |
| hc1428 | 2.157 | 31 | 1.33E-05 | 22 | 26.5 | 15 |
| hc1551 | 2.088 | 32 | 7.28E-06 | 44 | 21.4 | 44 |
| hc1404 | 2.086 | 33 | 1.93E-05 | 13 | 28.7 | 5 |
| hc1475 | 2.083 | 34 | 4.88E-06 | 56 | 22 | 37 |
| hc1484 | 1.898 | 35 | 1.94E-05 | 10 | 27.5 | 11 |
| hc1489 | 1.833 | 36 | 1.56E-05 | 19 | 25 | 24 |
| hc1603 | 1.802 | 37 | 5.08E-06 | 54 | 20.3 | 56 |
| hc1547 | 1.645 | 38 | 4.56E-06 | 59 | 19 | 68 |
| hc1584 | 1.636 | 39 | 1.94E-05 | 11 | 27.5 | 12 |
| hc1420 | 1.627 | 40 | 1.15E-05 | 26 | 23.5 | 28 |
| hc1344 | 1.605 | 41 | 1.49E-06 | 78 | 18.5 | 75 |
| hc1569 | 1.6 | 42 | 5.58E-06 | 53 | 21 | 45 |
| hc1322 | 1.596 | 43 | 9.10E-06 | 35 | 23 | 30 |
| hc1528 | 1.555 | 44 | 4.96E-06 | 55 | 19 | 69 |
| hc1505 | 1.531 | 45 | 7.08E-06 | 46 | 22 | 38 |
| hc1530 | 1.52 | 46 | 7.20E-06 | 45 | 20.5 | 53 |
| hc1327 | 1.507 | 47 | 1.10E-05 | 27 | 18.5 | 76 |
| hc1621 | 1.48 | 48 | 1.05E-05 | 30 | 23 | 31 |
| hc1474 | 1.465 | 49 | 4.81E-06 | 57 | 18.9 | 71 |
| hc1544 | 1.458 | 50 | 4.09E-06 | 62 | 19.8 | 59 |
| hc1351 | 1.423 | 51 | 3.09E-06 | 69 | 19.3 | 66 |
| hc1575 | 1.217 | 52 | 1.47E-06 | 79 | 20 | 58 |
| hc1332 | 1.169 | 53 | 2.71E-06 | 70 | 20.3 | 57 |
| hc1422 | 1.085 | 54 | 8.47E-06 | 39 | 22 | 39 |
| hc1524 | 1.026 | 55 | 4.53E-06 | 60 | 22.2 | 36 |
| hc1508 | 1.017 | 56 | 1.05E-05 | 29 | 22.7 | 33 |
| hc1517 | 0.938 | 57 | -1.17E-06 | 95 | 18 | 80 |
| hc1432 | 0.93 | 58 | 2.09E-06 | 74 | 18.8 | 72 |
| hc1440 | 0.897 | 59 | 4.70E-07 | 85 | 18.8 | 73 |
| hc1519 | 0.875 | 60 | 8.27E-06 | 40 | 18 | 81 |
| hc1545 | 0.789 | 61 | 3.16E-06 | 67 | 19.7 | 62 |
| hc1589 | 0.782 | 62 | 6.48E-06 | 47 | 21 | 46 |
| hc1578 | 0.764 | 63 | 8.15E-06 | 41 | 20.4 | 54 |
| hc1346 | 0.748 | 64 | -3.09E-06 | 106 | 16.2 | 101 |
| hc1405 | 0.628 | 65 | 1.04E-05 | 31 | 25.5 | 18 |
| hc1609 | 0.57 | 66 | 3.51E-06 | 66 | 17.2 | 90 |
| hc1411 | 0.548 | 67 | -4.29E-06 | 113 | 15.6 | 113 |
| hc1557 | 0.468 | 68 | 1.99E-06 | 77 | 22.5 | 35 |
| hc1402 | 0.446 | 69 | 1.32E-05 | 23 | 25.5 | 19 |
| hc1617 | 0.392 | 70 | 1.50E-07 | 86 | 19.5 | 63 |
| hc1529 | 0.292 | 71 | 5.00E-07 | 83 | 18 | 82 |
| hc1512 | 0.284 | 72 | 4.41E-06 | 61 | 20.4 | 55 |
| hc1501 | 0.279 | 73 | 2.24E-06 | 73 | 22 | 40 |
| hc1433 | 0.278 | 74 | 2.07E-06 | 75 | 23 | 32 |
| hc1535 | 0.201 | 75 | -3.00E-08 | 88 | 16.3 | 100 |
| hc1407 | 0.179 | 76 | -2.90E-06 | 105 | 17.3 | 88 |
| hc1610 | 0.134 | 77 | 2.26E-06 | 72 | 17.2 | 91 |
| hc1604 | 0.125 | 78 | 1.02E-06 | 81 | 17 | 92 |
| hc1549 | 0.117 | 79 | 4.68E-06 | 58 | 14.3 | 134 |
| hc1566 | 0.042 | 80 | -1.45E-06 | 96 | 19.4 | 65 |
| hc1447 | 0.013 | 81 | 1.24E-06 | 80 | 16 | 102 |
| hc1444 | 0.01 | 82 | 3.14E-06 | 68 | 16.7 | 96 |
| hc1342 | 0.005 | 83 | -3.49E-06 | 109 | 16 | 103 |
| hc1456 | -0.066 | 84 | -6.58E-06 | 126 | 15 | 119 |
| hc1500 | -0.081 | 85 | 9.57E-06 | 34 | 21 | 47 |
| hc1469 | -0.106 | 86 | -7.41E-06 | 130 | 13.5 | 145 |
| hc1325 | -0.107 | 87 | -9.80E-07 | 92 | 17.7 | 85 |
| hc1352 | -0.215 | 88 | -7.56E-06 | 135 | 17.6 | 87 |
| hc1550 | -0.253 | 89 | 1.00E-07 | 87 | 17.8 | 84 |
| hc1425 | -0.254 | 90 | 6.08E-06 | 49 | 20.8 | 50 |
| hc1608 | -0.265 | 91 | -7.33E-06 | 128 | 16.7 | 97 |
| hc1615 | -0.311 | 92 | -2.87E-06 | 104 | 17 | 93 |
| hc1521 | -0.312 | 93 | -3.25E-06 | 107 | 19 | 70 |
| hc1504 | -0.323 | 94 | -1.10E-06 | 93 | 18.5 | 77 |
| hc1452 | -0.333 | 95 | -5.49E-06 | 117 | 15 | 120 |
| hc1498 | -0.357 | 96 | 1.02E-05 | 33 | 22 | 41 |
| hc1553 | -0.376 | 97 | -2.50E-06 | 101 | 16.5 | 98 |
| hc1426 | -0.38 | 98 | 5.83E-06 | 52 | 18.2 | 79 |
| hc1525 | -0.401 | 99 | -1.65E-06 | 98 | 14.7 | 125 |
| hc1449 | -0.409 | 100 | -8.08E-06 | 138 | 16.5 | 99 |
| hc1417 | -0.426 | 101 | -1.29E-05 | 169 | 13 | 150 |
| hc1340 | -0.428 | 102 | -2.17E-06 | 100 | 16 | 104 |
| hc1572 | -0.436 | 103 | -1.50E-06 | 97 | 17 | 94 |
| hc1577 | -0.454 | 104 | -9.00E-07 | 91 | 17.3 | 89 |
| hc1341 | -0.462 | 105 | -4.09E-06 | 112 | 15.7 | 111 |
| hc1473 | -0.474 | 106 | 1.99E-06 | 76 | 18 | 83 |
| hc1602 | -0.516 | 107 | -6.34E-06 | 124 | 14.5 | 127 |
| hc1466 | -0.55 | 108 | -5.49E-06 | 116 | 13 | 151 |
| hc1522 | -0.6 | 109 | -6.56E-06 | 125 | 14.4 | 132 |
| hc1413 | -0.618 | 110 | -1.44E-05 | 180 | 12 | 162 |
| hc1568 | -0.628 | 111 | -8.00E-08 | 89 | 18.4 | 78 |
| hc1614 | -0.64 | 112 | -5.51E-06 | 119 | 13.9 | 139 |
| hc1443 | -0.668 | 113 | -3.52E-06 | 111 | 16 | 105 |
| hc1348 | -0.698 | 114 | -7.38E-06 | 129 | 13.8 | 141 |
| hc1345 | -0.72 | 115 | -1.92E-06 | 99 | 14.4 | 133 |
| hc1461 | -0.731 | 116 | -1.07E-05 | 155 | 13.2 | 149 |
| hc1539 | -0.755 | 117 | -1.10E-05 | 158 | 15.3 | 118 |
| hc1611 | -0.772 | 118 | -7.68E-06 | 136 | 13.9 | 140 |
| hc1451 | -0.782 | 119 | -7.56E-06 | 134 | 15.4 | 117 |
| hc1401 | -0.785 | 120 | 3.96E-06 | 63 | 21 | 48 |
| hc1558 | -0.848 | 121 | 3.89E-06 | 64 | 16.9 | 95 |
| hc1448 | -0.865 | 122 | -8.75E-06 | 143 | 14.5 | 128 |
| hc1534 | -0.909 | 123 | -5.76E-06 | 120 | 14.5 | 129 |
| hc1479 | -0.93 | 124 | -1.50E-07 | 90 | 15.7 | 112 |
| hc1439 | -0.943 | 125 | -6.31E-06 | 123 | 15.9 | 110 |
| hc1324 | -0.95 | 126 | -2.87E-06 | 103 | 15 | 121 |
| hc1480 | -0.959 | 127 | -3.49E-06 | 110 | 16 | 106 |
| hc1506 | -0.959 | 128 | -1.13E-06 | 94 | 17.7 | 86 |
| hc1527 | -0.99 | 129 | -2.72E-06 | 102 | 14.5 | 130 |
| hc1531 | -1.014 | 130 | -7.28E-06 | 127 | 15.5 | 114 |
| hc1414 | -1.146 | 131 | -1.06E-05 | 154 | 11 | 179 |
| hc1468 | -1.163 | 132 | -3.39E-06 | 108 | 10.2 | 188 |
| hc1526 | -1.193 | 133 | -8.20E-06 | 139 | 13 | 152 |
| hc1472 | -1.209 | 134 | -7.73E-06 | 137 | 14 | 135 |
| hc1445 | -1.37 | 135 | -1.03E-05 | 152 | 12.6 | 156 |
| hc1462 | -1.469 | 136 | -9.33E-06 | 146 | 13 | 153 |
| hc1470 | -1.493 | 137 | -8.31E-06 | 140 | 12.9 | 154 |
| hc1616 | -1.501 | 138 | -1.19E-05 | 162 | 14.7 | 126 |
| hc1548 | -1.511 | 139 | -1.27E-05 | 167 | 11.4 | 174 |
| hc1412 | -1.52 | 140 | -1.14E-05 | 159 | 12.5 | 157 |
| hc1619 | -1.552 | 141 | -5.96E-06 | 122 | 14.5 | 131 |
| hc1460 | -1.555 | 142 | -1.29E-05 | 170 | 13.3 | 148 |
| hc1408 | -1.585 | 143 | 5.00E-07 | 84 | 19.5 | 64 |
| hc1613 | -1.605 | 144 | -1.25E-05 | 164 | 11.5 | 173 |
| hc1465 | -1.642 | 145 | -1.35E-05 | 172 | 11 | 180 |
| hc1546 | -1.66 | 146 | -7.48E-06 | 132 | 14 | 136 |
| hc1435 | -1.703 | 147 | -7.53E-06 | 133 | 13.5 | 146 |
| hc1415 | -1.704 | 148 | -1.25E-05 | 165 | 10.8 | 185 |
| hc1446 | -1.71 | 149 | 2.42E-06 | 71 | 16 | 107 |
| hc1565 | -1.739 | 150 | -8.33E-06 | 141 | 11.7 | 170 |
| hc1421 | -1.8 | 151 | 7.20E-07 | 82 | 19.8 | 60 |
| hc1458 | -1.808 | 152 | -1.49E-05 | 182 | 12.7 | 155 |
| hc1416 | -1.811 | 153 | -1.55E-05 | 183 | 11.3 | 176 |
| hc1467 | -1.878 | 154 | -1.40E-05 | 174 | 11.8 | 168 |
| hc1471 | -1.915 | 155 | -9.05E-06 | 144 | 12.4 | 161 |
| hc1564 | -1.936 | 156 | -1.59E-05 | 184 | 12.5 | 158 |
| hc1418 | -2.006 | 157 | -1.31E-05 | 171 | 11.2 | 177 |
| hc1574 | -2.025 | 158 | -7.48E-06 | 131 | 14 | 137 |
| hc1573 | -2.036 | 159 | -9.90E-06 | 148 | 15 | 122 |
| hc1556 | -2.246 | 160 | -1.24E-05 | 163 | 11 | 181 |
| hc1532 | -2.252 | 161 | -1.48E-05 | 181 | 10.5 | 186 |
| hc1567 | -2.263 | 162 | -1.10E-05 | 157 | 11.7 | 171 |
| hc1343 | -2.265 | 163 | -9.93E-06 | 149 | 11.9 | 166 |
| hc1507 | -2.401 | 164 | -9.95E-06 | 151 | 16 | 108 |
| hc1518 | -2.419 | 165 | -1.07E-05 | 156 | 15.5 | 115 |
| hc1434 | -2.451 | 166 | -4.74E-06 | 114 | 16 | 109 |
| hc1523 | -2.489 | 167 | -5.86E-06 | 121 | 15 | 123 |
| hc1419 | -2.495 | 168 | -8.38E-06 | 142 | 13.8 | 142 |
| hc1570 | -2.532 | 169 | -1.15E-05 | 160 | 13.5 | 147 |
| hc1605 | -2.541 | 170 | -1.41E-05 | 176 | 13.7 | 143 |
| hc1441 | -2.544 | 171 | -1.37E-05 | 173 | 12.5 | 159 |
| hc1326 | -2.565 | 172 | -1.19E-05 | 161 | 11.8 | 169 |
| hc1347 | -2.675 | 173 | -1.41E-05 | 175 | 11.1 | 178 |
| hc1555 | -2.675 | 174 | -1.59E-05 | 185 | 11 | 182 |
| hc1350 | -2.981 | 175 | -1.29E-05 | 168 | 11.9 | 167 |
| hc1560 | -3.039 | 176 | -9.50E-06 | 147 | 14 | 138 |
| hc1576 | -3.092 | 177 | -9.25E-06 | 145 | 11.4 | 175 |
| hc1579 | -3.106 | 178 | -5.16E-06 | 115 | 15 | 124 |
| hc1349 | -3.16 | 179 | -1.42E-05 | 177 | 11.7 | 172 |
| hc1464 | -3.399 | 180 | -1.63E-05 | 186 | 11 | 183 |
| hc1438 | -3.515 | 181 | -1.26E-05 | 166 | 12 | 163 |
| hc1514 | -3.557 | 182 | -9.95E-06 | 150 | 13.7 | 144 |
| hc1533 | -3.631 | 183 | -1.68E-05 | 187 | 10.4 | 187 |
| hc1409 | -3.714 | 184 | -5.49E-06 | 118 | 15.5 | 116 |
| hc1476 | -3.737 | 185 | -1.05E-05 | 153 | 12.5 | 160 |
| hc1509 | -3.812 | 186 | -1.43E-05 | 179 | 12 | 164 |
| hc1442 | -4.125 | 187 | -1.81E-05 | 188 | 11 | 184 |
| hc1516 | -5.937 | 188 | -1.43E-05 | 178 | 12 | 165 |

Table S3. Predicted breeding values (PBV) of *Larix kaempferi* trees assigned as mother trees

| **Mother tree** | **PBV** | **Mother tree** | **PBV** | **Mother tree** | **PBV** | **Mother tree** | **PBV** |
| --- | --- | --- | --- | --- | --- | --- | --- |
| KW02 | 6.130 | KW22 | 1.626 | JPR-10 | -0.114 | KW48 | -1.660 |
| JB10 | 5.654 | KW35 | 1.606 | KW51 | -0.254 | KW13 | -1.970 |
| NO05 | 5.260 | KW40 | 1.480 | KW59 | -0.254 | GB04 | -2.006 |
| NO03 | 4.990 | CN19 | 1.458 | KW21 | -0.334 | KW44 | -2.024 |
| GB25 | 4.534 | KW04 | 1.422 | GB16 | -0.376 | JB03 | -2.086 |
| CN13 | 4.466 | KW08 | 1.306 | JPR-1 | -0.386 | JPR-12 | -2.150 |
| KW26 | 4.252 | KW52 | 1.278 | KW20 | -0.436 | KW42 | -2.226 |
| KW29 | 3.886 | KW24 | 1.174 | KW03 | -0.448 | GB31 | -2.240 |
| GB09 | 3.594 | KW57 | 1.086 | JPOR-1 | -0.454 | JN03 | -2.566 |
| JJ01 | 3.266 | JPR-4 | 1.062 | GB24 | -0.462 | JB09 | -2.988 |
| JB15 | 3.230 | GB05 | 0.930 | CN10 | -0.524 | KW58 | -3.236 |
| CN04 | 3.186 | KW10 | 0.928 | JB13 | -0.772 | GG01 | -3.438 |
| KW43 | 2.762 | JPR-3 | 0.854 | JB05 | -0.836 | GB21 | -3.484 |
| KW30 | 2.614 | JB11 | 0.782 | KW19 | -0.872 | KW07-2 | -3.576 |
| KW23 | 2.562 | GB11 | 0.748 | KW55 | -0.958 | KW07-1 | -3.584 |
| KW06 | 2.532 | JB14 | 0.580 | JB01 | -1.076 | JN04 | -3.706 |
| CN14 | 2.304 | GB12 | 0.548 | KW28 | -1.276 | GB13 | -3.738 |
| CN09 | 2.210 | GB29 | 0.516 | KW54 | -1.37 | CN05 | -4.044 |
| NO02 | 2.088 | NO06 | 0.502 | KW34 | -1.41 | JN01 | -4.338 |
| KW09 | 2.006 | JPR-8 | 0.292 | GB27 | -1.446 | KW56 | -4.608 |
| KW12 | 1.898 | CN16 | 0.270 | JB12 | -1.51 | CN15 | -5.276 |
| CB06 | 1.802 | CB03 | 0.004 | KW38 | -1.604 | JB08 | -5.736 |
| KW31 | 1.636 | JPR-9 | -0.018 | KW15 | -1.642 | GB23 | -7.800 |

| 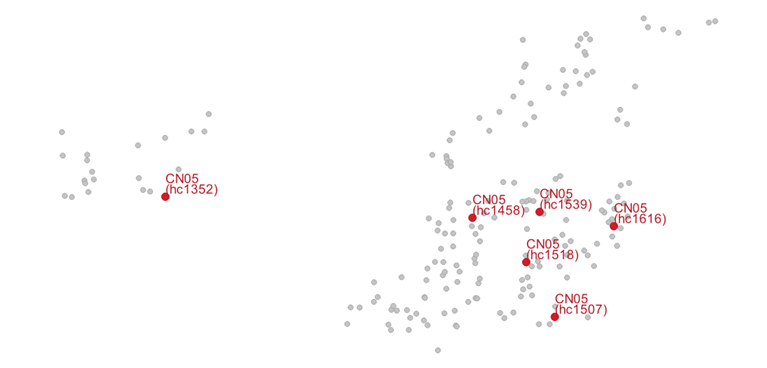 |
| --- |
| 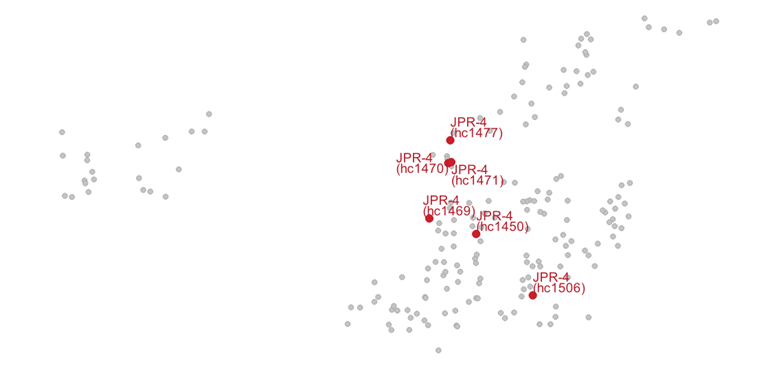 |
| 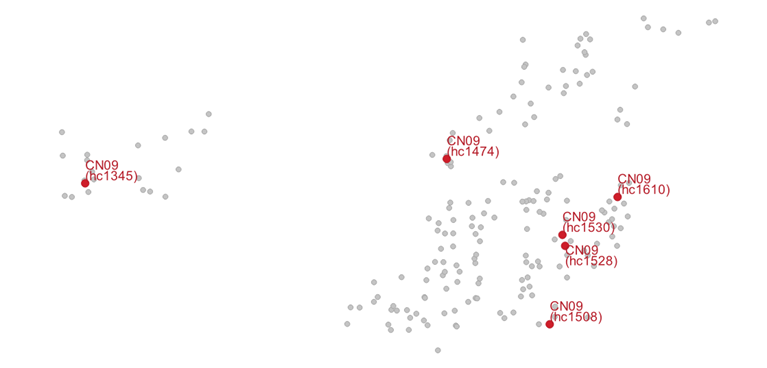 |

Fig S1. Locations of the progenies of mother trees with six assigned progenies

| (a)  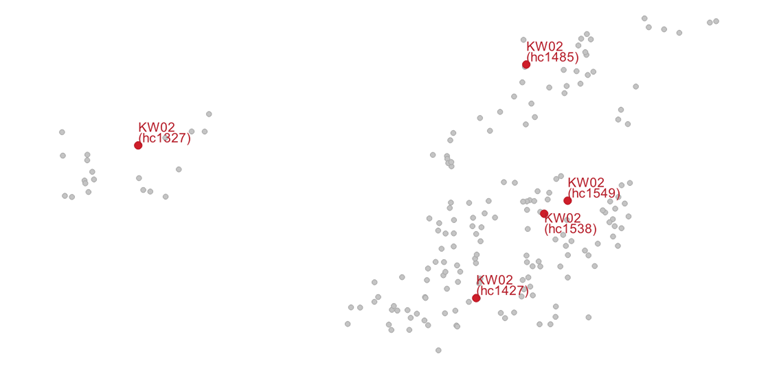 |
| --- |
| 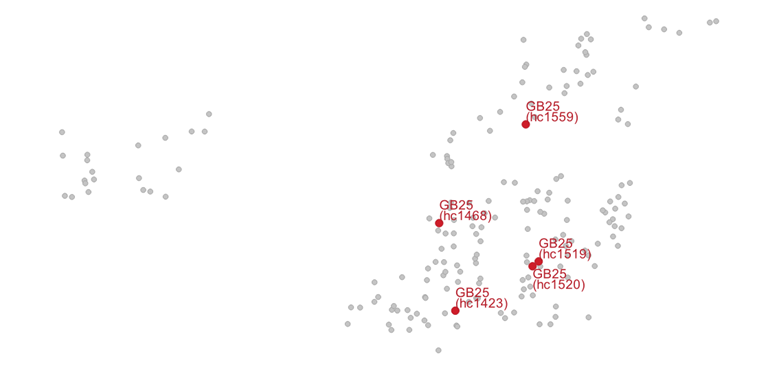 |
| 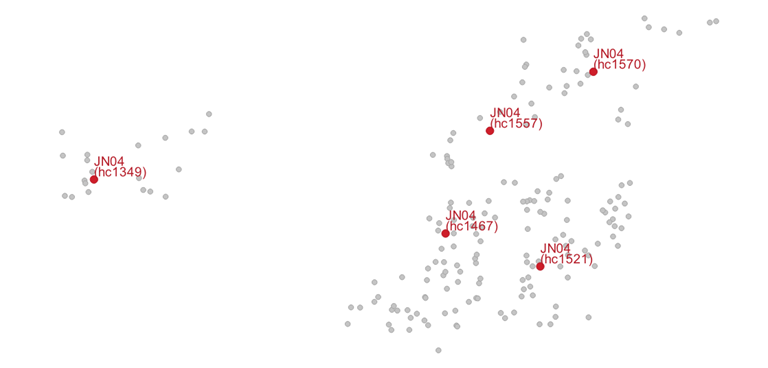 |
| (b)  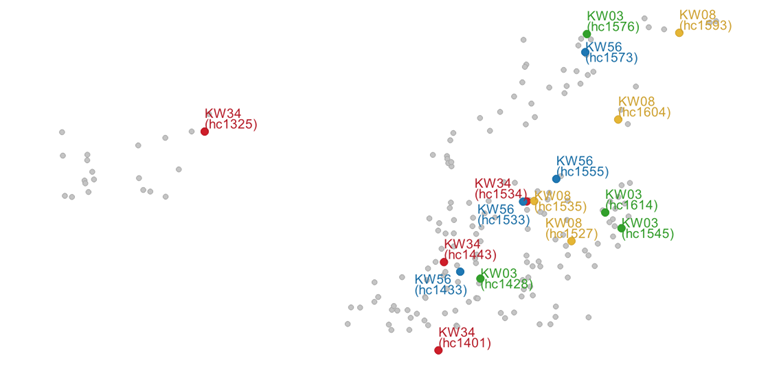 |

Fig S2. Locations of the progenies of mother trees with (a) five assigned progenies, and

(b) four assigned progenies

| 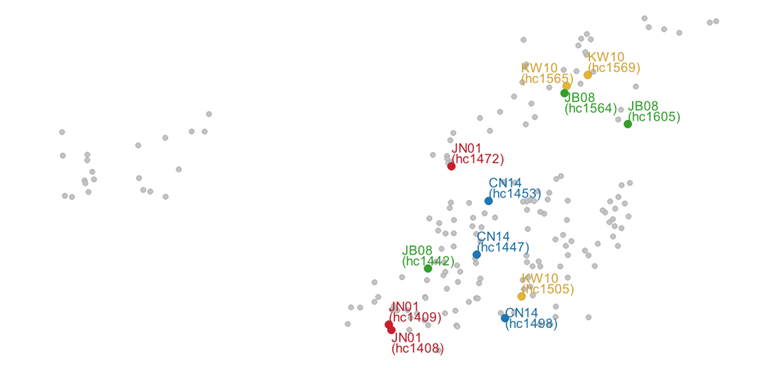 |
| --- |
| 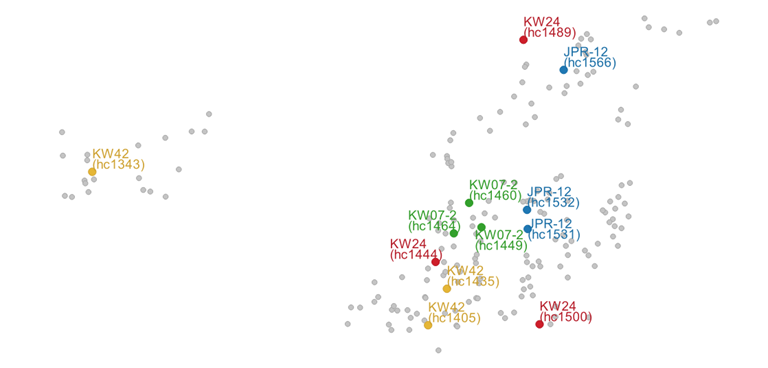 |
| 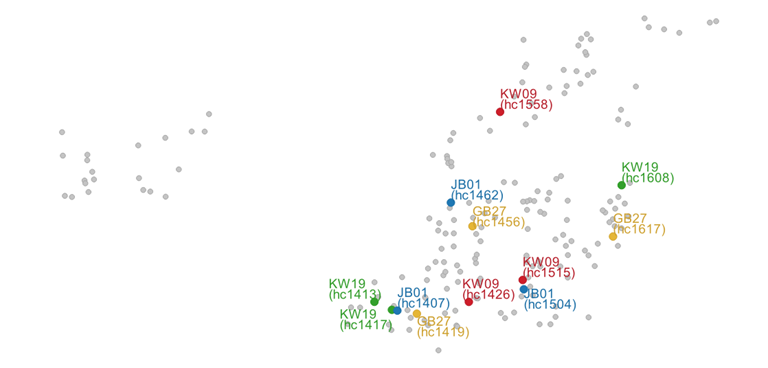 |
| 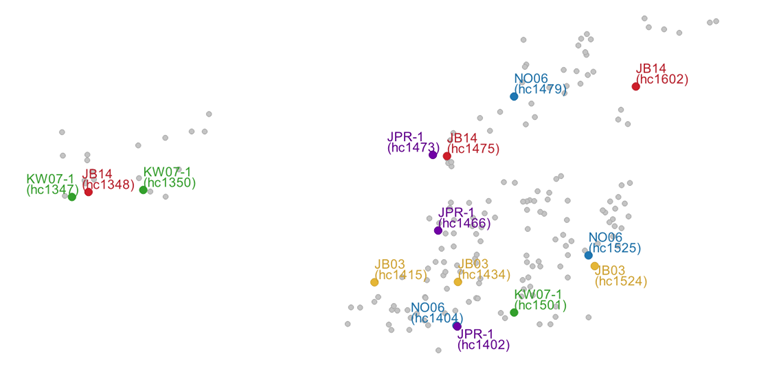 |

Fig S3. Locations of the progenies of mother trees with three assigned progenies

| 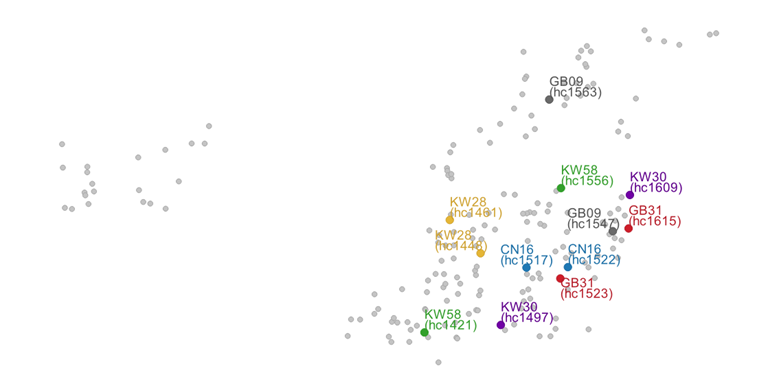 |
| --- |
| 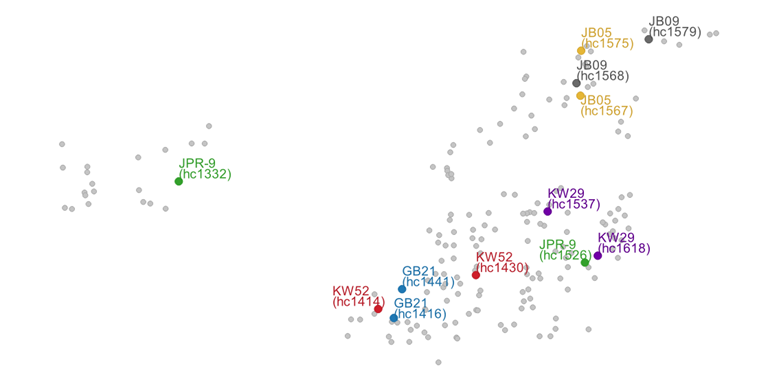 |
| 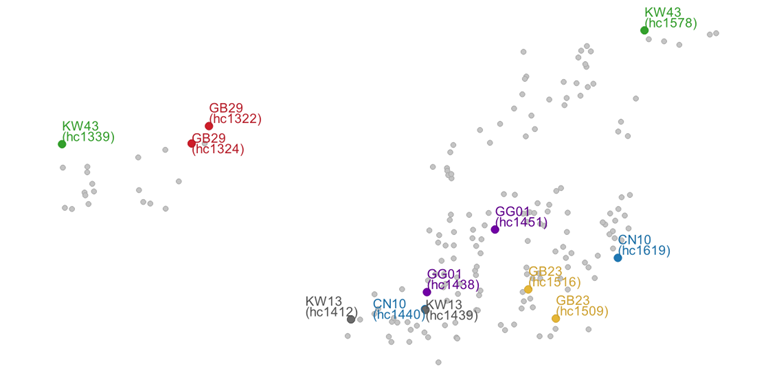 |
| 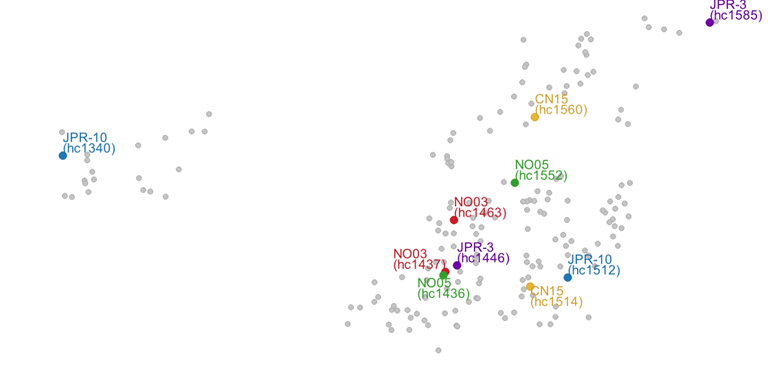 |

Fig S4. Locations of the progenies of mother trees with two assigned progenies


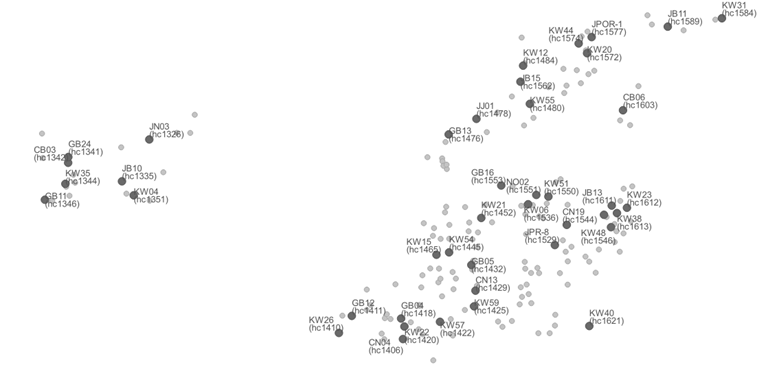


Fig S5. Location of the progenies of mother trees with one assigned progeny
